# Notes and News

**Published:** 1904-03-05

**Authors:** 


### Notes and News.

The King has promised a contribution of a hundred guineas to the fund now being raised for the establishment of a central institute of Medical Science in connection with London University, should the scheme be carried out.

H.R.H. the Princess of Wales, accompanied by the Bishop of London, the Countess of Errol, and Captain Holford, equerry in waiting, boldly plunged into the heart of the Ghetto on Thursday evening last. The goal was none other than the Whitechapel Rowton House in Fieldgate Street. At the entrance the Princess was received by the Superintendent, Mr. Brownlow, who at once proceeded to show the distinguished visitors over the building. The "Park," as the quadrangle dotted with pots of shrubs is called was the first spot peeped at through the gloom and then the big library was entered and traversed from end to end. As the party passed along the room through the cloud of tobacco smoke little or no attention was aroused. In fact the visit was a surprise one and hardly anybody among the 700 lodgers suspected who was there. An inspection of the vast dining-hall next followed, and even the scullery where the residents wash their platter and procure hot water for their tea was not neglected. All details here, in the cobbler's shop, or in the avenues of lockers where the lodgers keep their effects were briefly explained by Mr. Brownlow to her Royal Highness, who evinced a keen interest in everything. Finally, after signing the visitors' book, the Princess left, none the worse we may be sure for having, with Dr. Ingram's help as cicerone on the occasion, had a glimpse of how some of the King's humbler subjects take their ease in a poor man's hotel.

Prince Francis of Teck has joined the Weekly Board of Middlesex Hospital, of which Lord Sandhurst has been reappointed chairman and Sir Arthur T. Watson, K.C., vice-chairman.

The late Mrs. Mary Chrimes, of Moorgate Grange, Rotherham, who died on January 13th, bequeathed £1,000 each to the Devonshire Hospital, at Buxton, the Scarborough Hospital, the Sheffield Infirmary, the Rotherham Hospital, and the Jessop Hospital for Women, Sheffield.

Mr. Owen Jones, of Montague Mansions, who died on January 20th, bequeathed £2,000 each to the General Hospital at St. John, New Brunswick, and the Hospital of St. Stephen at New Brunswick.

Mrs. Mary Ann Irwin, who died at Brighton on January 17th, bequeathed £1,000 each to the Home for Incurables, Streatham Common; the Hospital for Sick Children, Great Ormond Street, Bloomsbury; and the Leicester Infirmary.

At the annual meeting of the subscribers to the Manchester Northern Hospital last week, one of the speakers, referring to the fact that it had been found necessary to realise some of the invested funds in order to clear off an adverse balance of about £1,000, said that "to sell investments was almost the policy of despair, but it was a less evil than to curtail the great work the hospital was doing."

The sum of £3,000 has been left to the General Hospital, Northampton, by Mr. W. W. Slye, of Thorndale House, West Haddon, who died in November last. Mr. Slye also bequeathed £2,000 to the Sussex County Hospital, £1,000 to the Northamptonshire Asylum at Berrywood, £1,000 to the Princess Alice Hospital at Eastbourne, and £250 to the Hospital for the Blind at Brighton.

At a conference, held last week, of the National Association for promoting the Welfare of the Feeble-Minded, Dr. Francis Warner showed how it was possible to train feeble-minded children by movements or muscular sense, and claimed that the most educative agents acting on such children were the teacher's body, hands, face, and voice. He described the various manual exercises which he advocated for their training, and said that they could educate the brain generally by the impressions the children received through their hands.

The death of Mr. John Thomson, chairman of the Manchester Royal Infirmary Board, took place on Sunday, and when the board met on Monday as usual, they adjourned without transacting any business in order to mark their sympathy with his widow and family. Mr. Thomson was one of the few old members of the board of management who were re-elected when the board was newly constituted to carry out the views of the trustees in favour of rebuilding the institution on some other than the Piccadilly site, and he was at once chosen chairman.

By the death of Sir Edward Sieveking, whose funeral took place on Saturday last, the medical profession has lost one of its most distinguished members. Though a Londoner by birth, he graduated at Edinburgh, becoming M.D. of that university in 1841. Shortly afterwards he settled in London to practise, becoming a Fellow of the Royal College of Physicians of London in 1852. He soon obtained a post on the visiting staff of St. Mary's Hospital, and also became physician to the National Hospital for the Paralysed and Epileptic, and the Lock Hospital. He was author of many notable medical works, including several important contributions on the subject of nervous diseases. In 1886 Dr. Sieveking received the honour of knighthood, and was appointed Physician Extraordinary to the King in 1901.

